# Effective treatment for prevention of post-operative adhesion after strabismus surgery in experimental rabbit model: 0.5 % tranilast ophthalmic solution

**DOI:** 10.1186/s12886-016-0344-8

**Published:** 2016-09-21

**Authors:** Sang Uk Choi, Kyoung Woo Kim, Nam Ju Moon

**Affiliations:** Department of Ophthalmology, College of Medicine, Chung-Ang University Hospital, 102, Heukseok-ro, Dongjak-gu, Seoul, 06973 Republic of Korea

**Keywords:** Tranilast, Strabismus surgery, Adhesion

## Abstract

**Background:**

Postoperative adhesion is one of the major complications of strabismus surgery and result in motility dysfunction that brings unpredictable surgical outcomes. However, there was no definitely accepted treatment method to prevent postoperative adhesion.

**Methods:**

A prospective, randomized, controlled experimental animal study was performed. Both eyes of each of 14 New Zealand White rabbits underwent superior rectus muscle recession. After the surgical procedure, the subjects were divided randomly into two groups; 0.5 % tranilast ophthalmic solutions and fluoroquinolone antibiotic eye drops were administered to the group tranilast (*N* = 14), while the group control (*N* = 14) received fluoroquinolone eye drops only. Five weeks after surgery, we evaluated gross adhesions with a numeric score (0 to 4). In addition, histopathologic examination with hematoxylin & eosin staining, Masson’s-trichrome staining, and anti-transforming growth factor beta 1 (TGF-β1) immuno-histochemical staining were done.

**Results:**

The group tranilast showed significantly less gross adhesion and inflammation than the group control (*P* = 0.01 and *P* < 0.001, respectively). Masson’s-trichrome staining revealed that post-operative collagen deposition was more prominent in the group control than the group tranilast (*P* < 0.001). Moreover, remarkable TGF-β1 expression was observed in areas with excessive collagen deposition.

**Conclusions:**

Instillation of 0.5 % tranilast ophthalmic solution is a simple and effective method for preventing post-operative adhesion after strabismus surgery.

## Background

Postoperative adhesion is one of the major complications of strabismus surgery and result in motility dysfunction that brings unpredictable surgical outcomes [1, 2]. Alleviating inflammation and thereby inhibiting the overproduction of extracellular matrix is essential to prevent fibrovascular proliferation and scar formation [3, 4]. Therefore, various surgical and medical approaches have been used to reduce postoperative inflammation and adhesion after strabismus surgery. These include antimetabolites (5-fluorouracil, mitomycin C), mechanical barriers (silicone sleeve, polyglactin 910 mesh, Seprafilm [Genzyme, Cambridge, MA, USA]), anti-inflammatory agents (triamcinolone), and lubricants (sodium hyaluronate) [1, 2, 5–10]. None of these, however, has been generally accepted because of associated complications, limitations or inconsistent outcomes [4].

Tranilast is a derivative of the amino acid tryptophan and was developed as an anti-allergy drug [11, 12]. It also prevents inflammation and collagen synthesis derived from keloid and hypertrophic scarring [11]. In addition, several in vitro and in vivo studies revealed that tranilast directly affected various parenchymal cells as anti-inflammatory and anti-fibrotic agent. Tranilast significantly reduced collagen synthesis, chemotaxis, cell migration, transforming growth factor beta 1 (TGF-β1) secretion, matrix metalloproteinase-1 secretion, and extracellular matrix production in various parenchymal cells [13–18].

In the ophthalmic field, tranilast eye drops are used to treat fibrotic ophthalmic diseases such as symblepharon, pterygium, and corneal haze after photorefractive keratectomy [19–21]. Prior study has demonstrated that direct slow-releasing tranilast could reduce adhesion and allowed delayed adjustment after surgery [22]. However, that is not a simple method to use in usual clinical setting. And, a long-term effect and safety of direct releasing system was not established in the wound healing. Also direct exposure of tranilast could induce stromal cell apoptosis [23].

With these backgrounds, the purpose of this study was to evaluate the anti-fibrotic effect of tranilast eye drops on experimental strabismus surgery in rabbit model, as measured by gross adhesion grading and histopathologic evaluation.

## Methods

A prospective, masked observer, controlled experiment was performed on adult female 14 New Zealand White rabbits (DooYeol Biotech, Seoul, Korea) weighing 2.0 to 3.0 kg. which housed in alone of given 2 weeks to acclimate to the housing facility. The rabbits were housed in individual mesh cages (0.90 × 0.65 × 0.50 m) hung at a height of 0.8 cm from the collecting trays and given access to rat maintenance food (Laboratory Rabbit Diet 5321, LabDiet, St. Louis, MO, USA) and water ad libitum. Environmental conditions were a temperature of 22 °C ±2°, humidity of 50 % ±10 %, lighting of 360 lx and a 12∶12 h light:dark cycle with lights on at 6:30 am and off at 6:30 pm. All experiments were conducted in accordance with the ARRIVE Guidelines for reporting animal research. And, study protocol was approved by Chung-Ang university institutional animal care and use committee (number 14-0043).

### Surgical procedure

We performed standard 3-mm recession with preserving Tenon’s capsule of the superior rectus muscle (SRM) strabismus surgery on both eyes in all rabbits. All surgeries were performed by the same surgeon (NJM). The animals were anesthetized with an intramuscular injection containing a mixture of tiletamine and zolazepam (Zoletil®, Virbac Lab, France) at 12.5 mg/kg, an aqueous solution of 2 % xylazine (Rompun®, Bayer Korea, Seoul, Korea) at 12.5 mg/kg, and topical anesthesia with 0.5 % proparacaine hydrochloride eye drops (Alcaine®, Alcon, Fort Worth, TX, USA). A 5 % povidone-iodine solution was applied to the inferior and superior fornix before surgery for antisepsis. The SRM was exposed through a limbal incision and was then isolated on a muscle hook. Three-millimeter recession was conducted with a double-armed 7-0 polyglactin suture (Vicryl®, Ethicon, Piscataway, NY, USA). The conjunctiva was then repositioned carefully and closed with two 7-0 polyglactin sutures in all eyes. Postoperatively, an antibiotic eye drop (Cravit®, Santen, Osaka, Japan) was used three times a day in one eye (*N* = 14) and an antibiotic eye drop and 0.5 % tranilast eye drops (Krix®, Choongwae Shin Yak, Seoul, Korea) were used three times a day in the contralateral eye (*N* = 14) which was randomly selected by using table of random numbers. All eye drops were administered 6:30 to 7:00, 12:30 to 13:00, and 18:30 to 19:00.

### Evaluation of gross adhesions

**Fig. 1:**
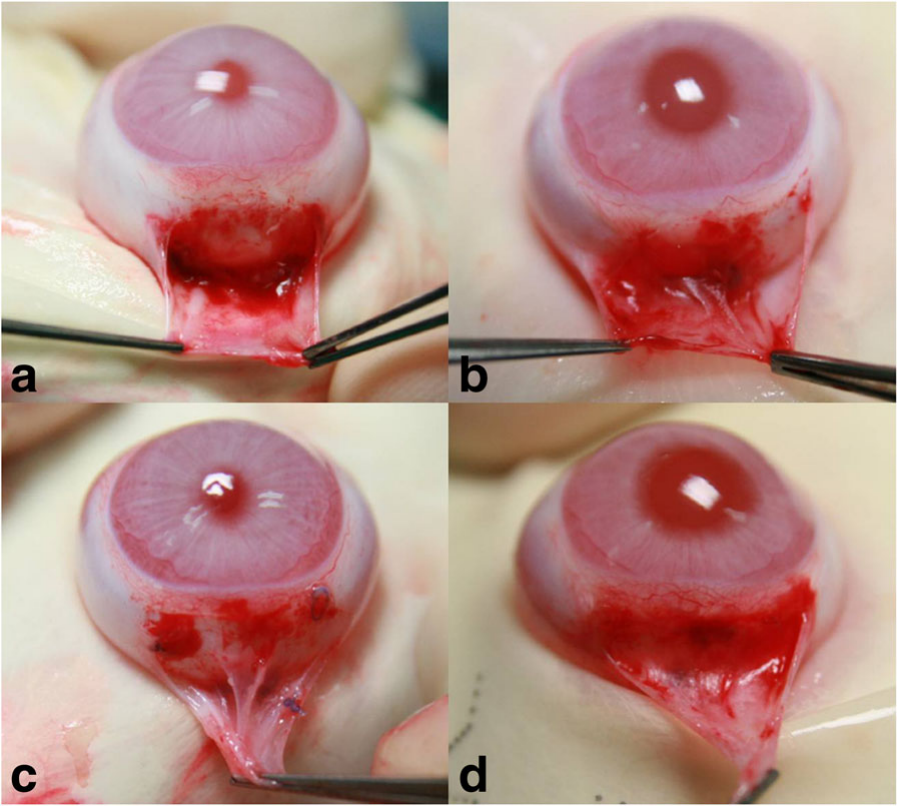
Reference photograph of the degree of adhesion. **a** Grade 1, filmy adhesion easily separable with blunt dissection; **b** grade 2, mild-to-moderate adhesion with a freely dissectible plane; **c** grade 3, moderate-to-dense adhesion with difficult dissection; **d** grade 4, non-dissectible plane

Five weeks after the surgery all rabbits were re-anesthetized. Then, gross adhesion grades of recession site were evaluated. The grades were classified by severity and were scored from 0 to 4 as follows: 0 = no adhesion; 1 = filmy adhesion easily separable with blunt dissection; 2 = mild-to-moderate adhesion with freely dissectible plane; 3 = moderate-to-dense adhesion with difficult dissection; and 4 = non-dissectible plane (Fig. 1).

### Histopathological examination

After adhesion grading, the eyes were enucleated beware of damage to the recession site. A two of 4 mm width incision was made parallel with recession at 2 mm anteriorly and 4 mm posteriorly to recession site. Then incision was extended to first two incisions to make histologic block. The histologic block fixed in 10 % formaldehyde and embedded in paraffin. Three sagittal sections for each block were made perpendicularly to the line of the postsurgical insertion of the SRM. Tissues in each group were examined microscopically using hematoxylin & eosin (H&E) staining to general histologic observation. Inflammation was quantified by averaging the number of inflammatory cells in three high powered field views on light microscopy of recession site stroma on each section. Masson’s-trichrome staining was performed to quantification of collagen formation, and TGF-β1 expression was identified using anti-TGF-β1 antibody (Goat anti-Rabbit IgG Antibody; Novusbio, Littleton, CO, USA) through immunohistochemical (IHC) staining.

### Quantification of fibrosis

Three sections of Masson’s-trichrome staining images of extraocular muscle at recession site (original magnification ×200), were obtained per each eye using the CCD camera, light microscope, and computer software (Zeiss AxioCam ICc1, Zeiss Axiskop40 microscope, and ZEN 2012 software, Zeiss Korea, Seoul, Korea). Then, the obtained images were imported into Adobe® Photoshop® CS5 software (Adobe Systems Inc, San Jose, CA, USA). Total pixel numbers of intermuscular collagen deposition of each section were recorded using polygonal lasso tool according to the previously published protocol to quantify the degree of fibrosis [24].

### Statistical analyses

Fisher’s exact test was used to determine the relationship between the severity of adhesion (Grade 0 to 4) and the application of tranilast eye drops. The Mann-Whitney test was used to compare degree of inflammation on H & E staining and amount of fibrosis on Masson’s-trichrome staining. Statistical analyses were performed with SPSS version 18.0 (SPSS, Inc., Chicago, IL, USA) software. The level of statistical significance was set at 5 % (*P* < 0.05) for all tests.

## Results

### Degree of gross adhesion

Eyes in group tranilast (14/28 eyes) showed filmy adhesion (score 1; 6 eyes) or mild-to-moderate adhesion (score 2; 7 eyes) except one which had moderate-to-dense adhesion (score 3) (Table 1). Whereas, the eyes in group control (14/28 eyes) revealed adhesion ranging from mild-to-moderate (score 2; 4 eyes) and moderate-to-dense (score 3; 8 eyes) to non-dissectible (score 4; 2 eyes). There was less post-operative recession site adhesion in group tranilast compared to group control (*P* = 0.01, Fisher’s exact test). There was no adverse event such as infection in the experimental and control groups.

**Table 1:** Numerical gross adhesion grades for each eye

Adhesion grade	0	1	2	3	4	Total *N* (%)
Control	0 (0 %)	0 (0 %)	4 (29 %)	8 (57 %)	2 (14 %)	14 (100 %)
Tranilast	0 (0 %)	6 (43 %)	7 (50 %)	1 (7 %)	0 (0 %)	14 (100 %)

### Histopathologic examination and quantitative analysis

**Fig. 2:**
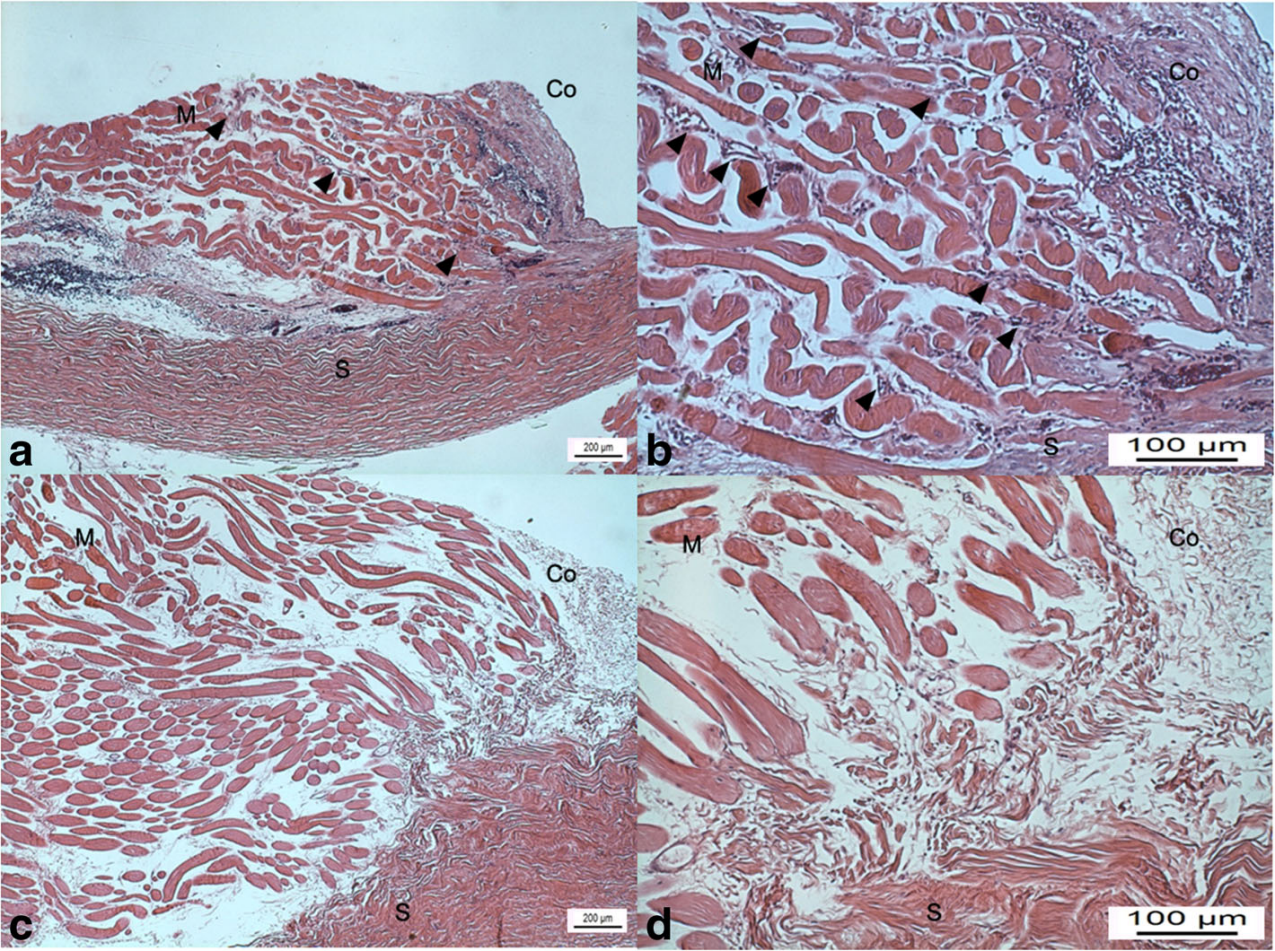
Light microscopic findings on hematoxylin and eosin staining of group control (**a**, **b**) and group tranilast (**c**, **d**). The group control shows more prominent fibrovascular proliferation in superior rectus muscle then the group tranilast (*arrow heads* in [**a**, **b**]). The group tranilast shows scant fibrosis and inflammatory cell infiltration (**c**, **d**; *left column*, original magnification ×40; *right column*, original magnification ×100; *M* superior rectus muscle, *S* sclera, *Co* Conjunctiva)

**Fig. 3:**
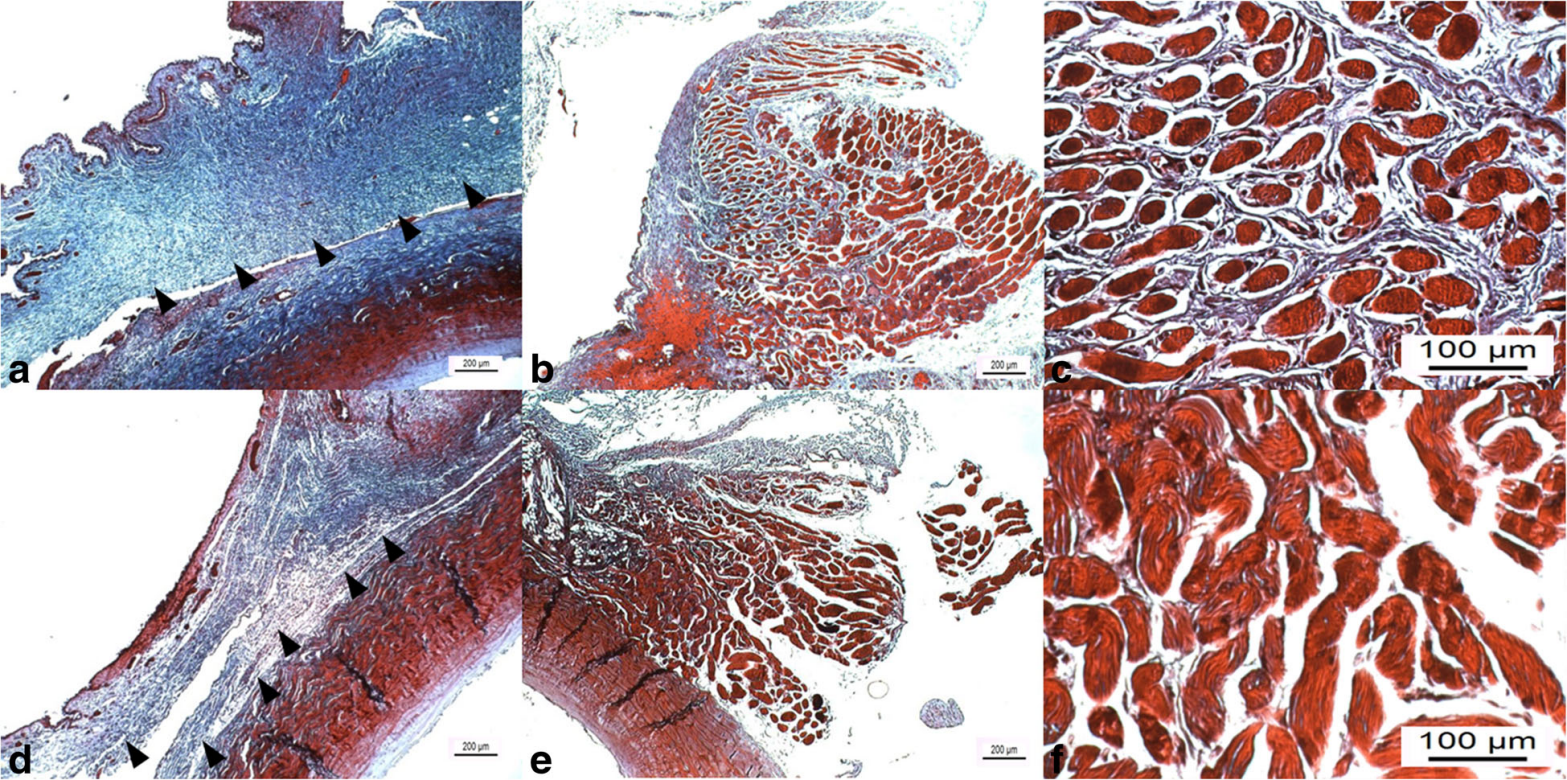
Light microscopic findings from Masson’s-trichrome staining of group control (**a**, **b**, **c**) and group tranilast (**d**, **e**, **f**). The group control shows more intense collagen deposition than group tranilast at limbal incision site (*arrow heads* in [**a**, **d**]). Also, there was a marked reduction in the density of superior rectus muscle fibrosis (**c**, **f**; **a**, **b**, **d**, **e**, original magnification ×40; **c**, **f**, original magnification ×200)

**Fig. 4:**
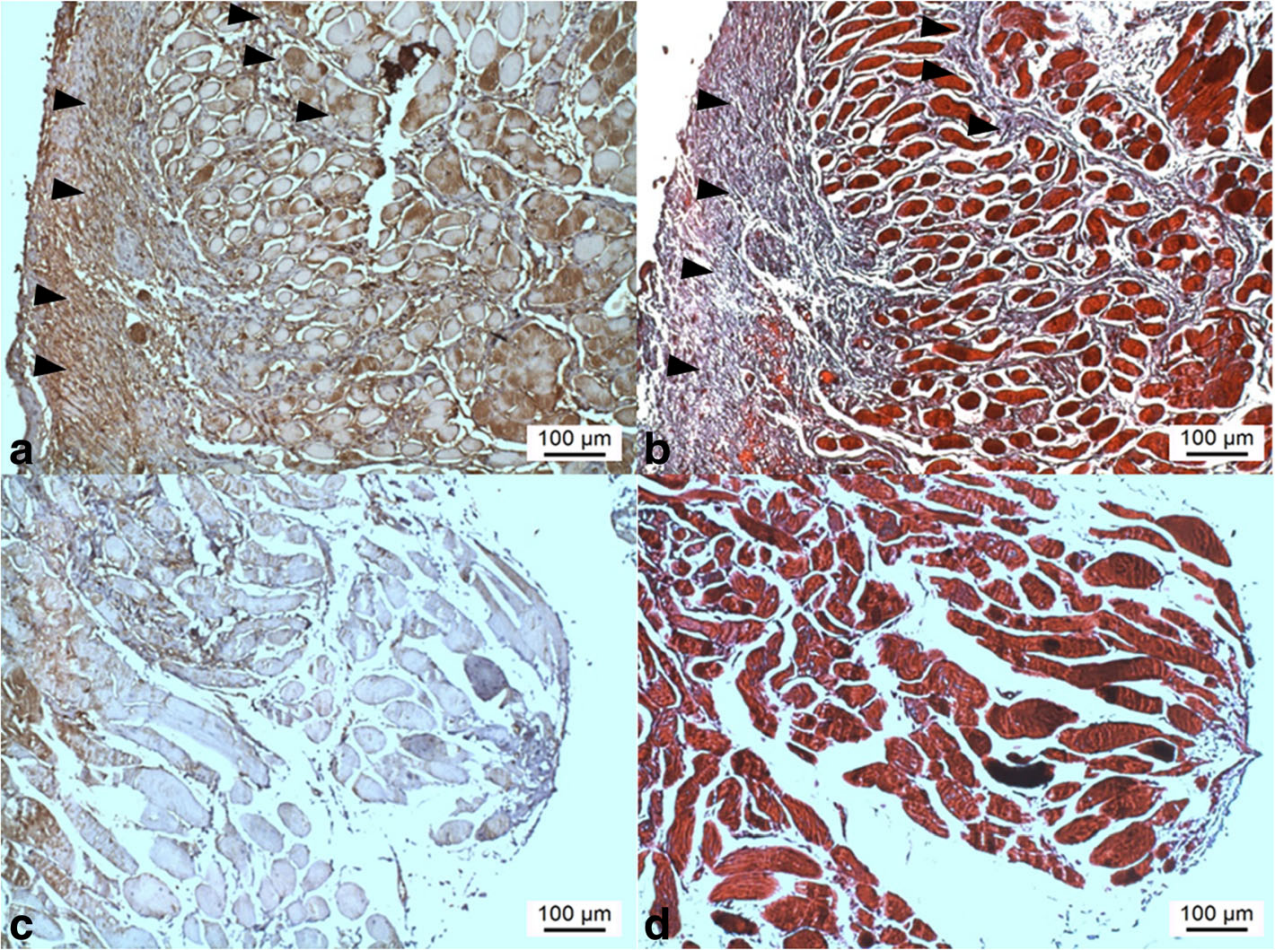
Light microscopic findings of immuno-histochemical staining of anti-transforming growth factor beta 1 (TGF-β1) (**a**, **c**) and Masson’s-trichrome staining (**b**, **d**) of the superior rectus muscle at identical sections (**a**, **b**, group control; **c**, **d**, group tranilast). Prominent TGF- β1 expression was observed in the area of excessive collagen deposition (*arrow heads* in [**a**, **b**]; **a**, **b**, **c**, **d**, original magnification ×100)

The group tranilast demonstrated less inflammation and fibrosis around the SRM than group control (Fig. 2). The group control presented significantly more inflammatory cells (mostly small lymphocytes) than the group tranilast (Table 2; *P* < 0.001, Mann-Whitney test). Masson’s trichrome staining revealed that group control presented more intense post-operative fibrosis than group tranilast (Fig. 3). Moreover, in quantitative analysis of fibrosis, there was a significant reduction of muscle fibrosis in the tranilast treated eyes compared with control eyes (Table 3; *P* < 0.001, Mann-Whitney test). The group control showed more prominent TGF-β1 expression than group tranilast (Fig. 4).

**Table 2:** Inflammatory cell count per high power field on light microscopy

	Cells/HPF (mean ± SD)	
Control	43.35 ± 12.25	*P* < 0.001*
Tranilast	23.35 ± 10.55	

*HPF* high power field, *SD* standards deviation

*Mann-Whitney test

**Table 3:** Quantitative analysis of fibrosis through Masson’s trichrome staining

	Number of pixels (mean ± SD)	
Control	133,798 ± 60,855	*P* < 0.001*
Tranilast	54,011 ± 26,873	

*SD* standards deviation

*Mann-Whitney test

## Discussion

Several studies were performed to prevent formation of adhesion and to alleviate motility dysfunction after strabismus surgery by using various materials and medical agents. The materials which include tissue implants such as amniotic membrane, silicone, supramid sleeve, polyglactin mesh sleeves [4, 25, 26]. However, various level of clinical success and foreign body reaction or extrusion has discouraging the purpose of their insertion [1]. Similarly, the medical agent such as mitomycin C, and 5-fluorouracil demonstrated positive results in reducing the postoperative adhesions and fibrosis [1, 7]. However, these agents have the risk for serious side effects such as corneoscleral melt [27]. More recently, monoclonal anti-vascular endothelial growth factor antibody has been investigated to reduce postoperative adhesions. But, it was insufficient to prevent postoperative adhesions [28].

The TGF-β superfamily consists of diversiform proteins that have pleiotropic function, including cell cycle control, chemotaxis, and wound healing [29, 30]. The TGF-β is well known as the crucial molecule in tissue organ fibrosis [31]. The TGF-β signaling induces various mesenchymal precursor cells to myofibroblast transformation which characterized α-smooth muscle actin expression and collagen secretion [32, 33]. TGF-β1, 2 and 3 are three isoforms exist in mammalian cells [34]. In human eye, it was previously reported that normal conjunctiva showed much weaker expression of TGF-β family than fibrous conjunctiva tissue [35]. Moreover, TGF-β1 is overexpressed in cultured fibroblasts from fibrous conjunctiva among three isoforms of TGF-β [36]. Therefore, TGF-β, especially TGF-β1, has been the treatment target to prevent the formation of postsurgical fibrosis and adhesions in strabismus surgery [37].

To our knowledge, there are no published reports on the histopathologic evaluation for the effect of tranilast eye drops after strabismus surgery. This study investigated the effects of tranilast eye drops on fibrosis and inflammation caused by extraocular muscle surgery in rabbits. Five weeks after operation, with 0.5 % tranilast eye drops, the group tranilast demonstrated less gross adhesion, inflammation and fibrosis than the group control.

Those findings are comparable to previous studies reporting that a slow-release tranilast system reduced postoperative adhesions in strabismus surgery [22]. Hwang et al. wrapped the extraocular muscle with a sheet of polytetrafluoroethylene/polylactide-co-glycolide laminate with tranilast, reducing adhesion during a delayed adjustment procedure [22]. This system is highly effective, as it directly and consistently releases the active agent. However, a direct slow-release system requires an additional complicated surgical procedure and has the problem of remained foreign body in surgical site with the case of standard strabismus surgery. We therefore used more simple postoperative management and found that tranilast eye drops were comparable to a direct release system in terms of postoperative adhesion and fibrosis.

Tranilast eye drops may decrease surgical site adhesion and fibrosis via anti-inflammatory and anti-fibrotic action. Tranilast eye drops showed favorable anti-inflammatory action as a cytokine modifier. It suppress the release of interleukin-2 and interleukin-1β from monocytes and macrophages, which are key cytokines that initiate inflammation [11]. Furthermore, tranilast inhibits the progression of inflammation; it inhibits chemotaxis by suppressing the expression of vascular cell adhesion protein-1 and intercellular adhesion molecule 1 [38–40]. Additionally, tranilast has inhibitory effect of collagen deposition by inhibiting fibroblast proliferation [41] and limiting TGF-β induced fibrosis and excessive formation of extracellular matrix inhibiting expression of TGF-β1 [42, 43]. This effect could be obtained even by application of eye drops and was confirmed using Masson’s-trichrome and IHC staining in the present study (Figs. 3 and 4).

The present study has some limitations. Surgical outcomes were evaluated at only one point, 5 weeks after surgery, to ensure completion of the proliferative phase, which includes collagen synthesis and adhesion formation [44]. This is suitable for evaluating fibrosis and surgical site adhesion, but earlier postoperative evaluation needs to clarify the role of tranilast in the acute inflammatory and proliferative phases of inflammation. In addition, it is necessary to investigate the effect of other anti-inflammatory and anti-fibrotic therapeutic agents, such as nonsteroidal anti-inflammatory drugs and steroids eye drops, compared to tranilast eye drops.

## Conclusions

In conclusion, 5-week instillation of 0.5 % tranilast eye drops is a simple and effective method for the prevention of post-operative adhesion and fibrosis after strabismus. This is the first experimental study to evaluate the effect of tranilast eye drops as a treatment for postoperative adhesion after strabismus surgery, through the downregulation of inflammation and fibrosis.

## Abbreviations

H & E: Hematoxylin & eosin
IHC: Immunohistochemical
SRM: Superior rectus muscle
TGF-β1: Transforming growth factor beta 1
